# Activating NKG2C Receptor: Functional Characteristics and Current Strategies in Clinical Applications

**DOI:** 10.1007/s00005-023-00674-z

**Published:** 2023-03-10

**Authors:** Jagoda Siemaszko, Aleksandra Marzec-Przyszlak, Katarzyna Bogunia-Kubik

**Affiliations:** 1 Laboratory of Clinical Immunogenetics and Pharmacogenetics, Hirszfeld Institute of Immunology and Experimental Therapy, Polish Academy of Sciences, Wroclaw, Poland; 2 Department of Biosensors and Processing of Biomedical Signals, Faculty of Biomedical Engineering, Silesian University of Technology, Zabrze, Poland; 3 Department of Biomedical Engineering, Faculty of Electrical Engineering and Communication, Brno University of Technology, Brno, Czech Republic

**Keywords:** NKG2C, NK cell receptors, NK cells, HLA-E

## Abstract

The interest in NK cells and their cytotoxic activity against tumour, infected or transformed cells continuously increases as they become a new efficient and off-the-shelf agents in immunotherapies. Their actions are balanced by a wide set of activating and inhibitory receptors, recognizing their complementary ligands on target cells. One of the most studied receptors is the activating CD94/NKG2C molecule, which is a member of the C-type lectin-like family. This review is intended to summarise latest research findings on the clinical relevance of NKG2C receptor and to examine its contribution to current and potential therapeutic strategies. It outlines functional characteristics and molecular features of CD94/NKG2C, its interactions with HLA-E molecule and presented antigens, pointing out a key role of this receptor in immunosurveillance, especially in the human cytomegalovirus infection. Additionally, the authors attempt to shed some light on receptor’s unique interaction with its ligand which is shared with another receptor (CD94/NKG2A) with rather opposite properties.

## NKG2C: An Activating Receptor of NK Cells

Natural killer (NK) cells, also known as large granular lymphocytes, constitute a subgroup of recently discovered innate lymphoid cells (ILCs), and together with the ILC1 subpopulation are responsible for type 1 immunity functions, e.g. natural cytotoxicity. Originally considered as components only of the innate immunity, the NK cells have also developed characteristics restrained for adaptive immunity (Pende et al. 2019; Vivier et al. 2011, 2018). The NK cells functions are regulated and properly balanced by a wide set of activating and inhibitory receptors (Quatrini et al. 2021). Among the activating receptors, the CD94/NKG2C remains to belong to the ones of considerable influence on NK functioning. The CD94/NKG2C is a heterodimeric receptor, consisted of two type II proteins and is a member of the C-type lectin-like family. Similar to the other activating NKG2 receptors, the NKG2C has a negatively charged residue (either a lysine or an arginine) in its transmembrane domain. Activation signal is transduced through the DAP-12 adaptor molecule, which contains immunoreceptor tyrosine-based activation motifs. The ligand for CD94/NKG2C is a non-classical HLA-E molecule of major histocompatibility (MHC) class I proteins. Its recognition is crucial for triggering the NK cell cytotoxicity and cytokine production (Glienke et al. 1998; Lanier et al. 1998; Miyashita et al. 2004). NK cell receptors can also be found on some T cells, such as CD8^+^ T lymphocytes, which express NKG2 receptors of activating (NKG2C, NKG2D) and inhibiting (NKG2A) properties (Patel et al. 2013). The CD94/NKG2C receptor may even operate like an alternative pathway to the T cell receptor in subset of NKG2C^+^ CD8^+^ T cells, triggering proliferation and effector functions of those cells (Gumá et al. 2005).

**Fig. 1:**
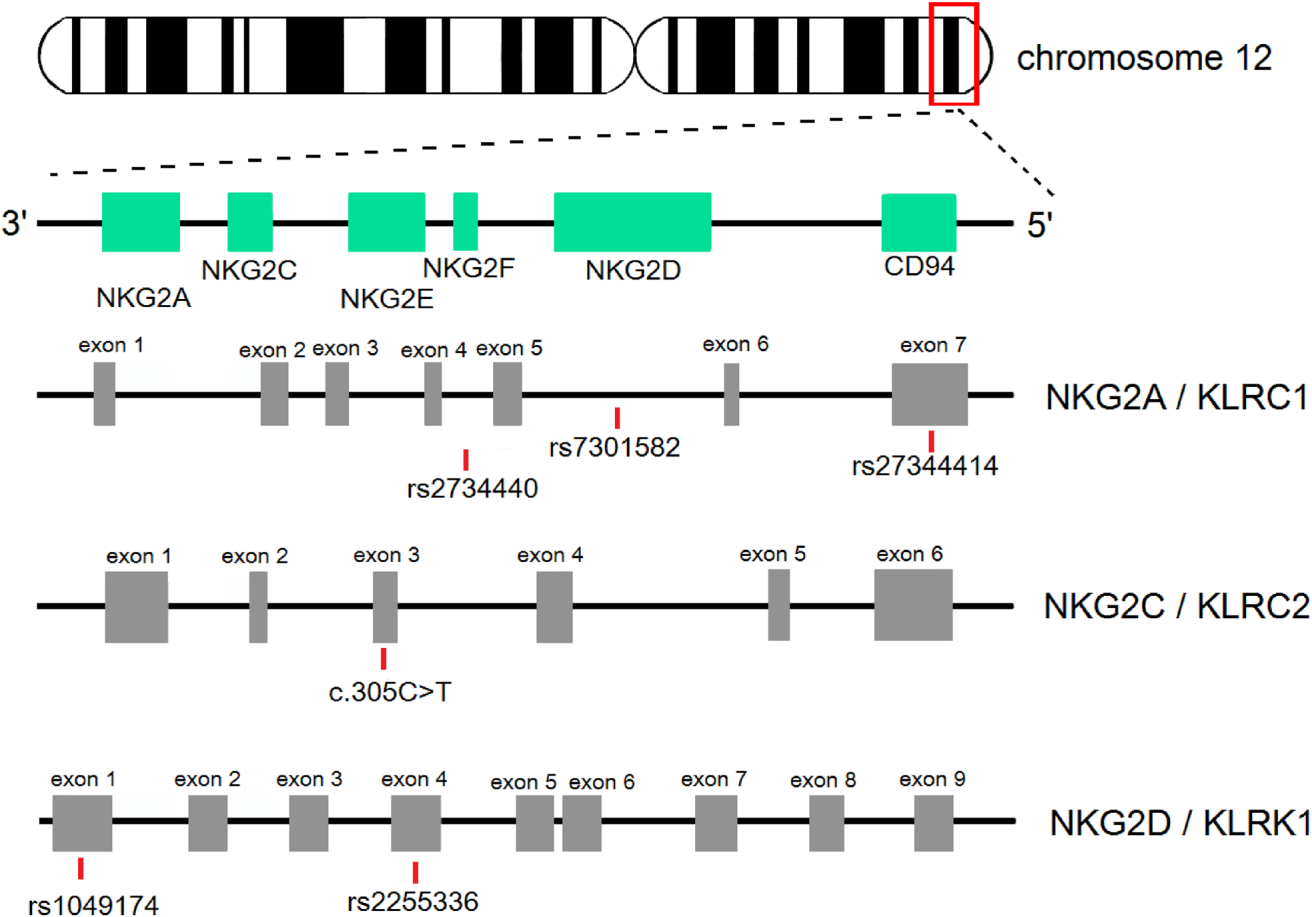
Genomic organization of the CD94/NKG2 region on chromosome 12. In the natural killer gene complex, the genes encoding for NKG2A, NKG2C, NKG2E, NKG2F and NKG2D receptors as well as for the CD94 molecule, are located on the short arm of chromosome. The most important single-nucleotide polymorphisms of major receptors are detailed

The CD94/NKG2C receptor is encoded by the 6 kpb length *NKG2C* gene, also referred as *KLRC2*, located within the NK complex on chromosome 12p12.3–13.2 (Fig. 1). It consists of six exons and five introns. Protein product has 231 amino acids in length and a molecular mass of 26,159 Da. The two homologous proteins of NKG2C are NKG2E and alternatively spliced NKG2H. Both *NKG2C* and *NKG2E* genes are highly identical (92.1%) at the genomic level, have duplicated 255-bp length DNA containing a second version of exon III (termed IIIB). The differences in gene sequence were found at the 3’ end of *NKG2E*, resulting in additional *Alu* amino acid. The 3’ region of *NKG2-A, -E* and *-F* receptor, rich in *Alu* repeats, is known as coding for the extracellular domain of the NKG2 receptors and may affect ligand specificity (Glienke et al. 1998). It is known that *Alu* sequences occur with a frequency of approximately one element per 4 kb in the human genome, and could cause abrogation of transcriptional activity (Brostjan et al. 2000).

It has been assumed that the NKG2 family is rather non-polymorphic although some variabilities in genomic sequences have been described. In our previous report on NKG2D (Siemaszko et al. 2021) we provided a comprehensive summary of the clinically associated genetic variations of its gene. As for the *NKG2C* gene, two single-nucleotide polymorphisms (SNPs): rs10587111 and rs10588530 have been identified recently in a set of genetic mutations occurred at higher frequency in patients infected with influenza A (H7N9) (Chen et al. 2016). There are three reported alleles of *NKG2C*. Two of them, named, respectively, *01 and *02 by Shum et al. (2002), were found in individuals of different ethnicities. They differ in two single-nucleotide non-synonymous polymorphisms (c.5G > A, Ser2Asn, and c.305C > T, Ser102Phe). The first one affects the cytoplasmic tail, while the second one is located in the stem connecting the transmembrane region with the ligand-binding domain. Third novel *03 allele, encoding asparagine 2, like NKG2C*02 and serine 102, like NKG2C*01, was recently discovered in two unrelated Caucasoids (Asenjo et al. 2021).

One of the most characteristic features of the *NKG2C* is its genomic mutation resulting in deletion of a ~ 16-kb region encompassing the gene, first observed in a Japanese population (Hikami et al. 2003; Miyashita et al. 2004). The evidence for *NKG2C* deletion was found due to the polymorphism screening (Hikami et al. 2003) and the breakpoint was detected within the 292‐bp region 1.5–1.8 kb telomeric from the 3’ untranslated region of the *NKG2A*. Deletion haplotype comes with a unique set of nucleotides around the breakpoint with several *Alu* repeats presented. Although the *NKG2C* deletion breakpoint is not located in the *Alu* sequence, the *Alu* repeats are usually involved in gene recombinations (Miyashita et al. 2004). The *NKG2C* gene homozygous ~ 16 kb deletion is distributed with frequency not exceeding 5% of studied populations (Hikami et al. 2003; Li et al. 2015; López-Botet et al. 2014; Miyashita et al. 2004; Moraru et al. 2012; Muntasell et al. 2013; Thomas et al. 2012; Toson et al. 2022). However, studies on West African populations from Gambia and Guinea-Bissau revealed that *NKG2C* deletion homozygosity occurred with almost 14% frequency (Goncalves et al. 2016; Goodier et al. 2014). This indicates that the *NKG2C* gene is not essential for survival and reproduction. Its deletion may be compensated possibly by the *NKG2E* gene (Hikami et al. 2003; Miyashita et al. 2004). The consequence of *NKG2C* deletion has been investigated in many studies, reviewed in the next paragraphs. The *NKG2C* wild-type (*wt*) allele has a protective effect for human cytomegalovirus (HCMV) viremia and the deletion (*del*) variant was associated with higher risk of HCMV viremia and disease development (Rangel-Ramírez et al. 2014). The high frequency of viremia and HCMV-disease was also observed after lung transplantation in patients with *del/del* homozygous genotype (Goodier et al. 2014; Vietzen et al. 2018). It was reported that patients carrying *del*/*del* genotype lacked NKG2C expression, while in *wt/del* heterozygotes the expression was intermediate (Muntasell et al. 2013). Furthermore, as reported by Muntasell et al. (2013), in NKG2C *wt/wt* HCMV^+^ individuals the receptor’s expression affects the NKG2C^bright^ NK cells by increasing their number. The *NKG2C* genotype also modulates HCMV-induced expansion of NKG2C^+^ cells. HCMV-seropositive *NKG2C wt*/*wt* children and adults express higher numbers of NKG2C^bright^ cells than hemizygous *NKG2C wt*/*del* subjects. Moreover, the quantitative differences in surface expression of NKG2C as well as in the response to receptor engagement were also noticed (López-Botet et al. 2014).

## Significance of Interaction Between HLA-E Molecule and its NKG2A and NKG2C Receptors

Being a member of class Ib non-classical human leukocyte antigens (HLA) molecules, HLA-E is expressed constitutively on B and T lymphocytes, NK cells, monocytes, trophoblasts, and also in tumour cells (Coupel et al. 2007). The *HLA-E*-encoding gene is located in chromosome 6. It is well known that *HLA-E* is not as polymorphic as the other HLA molecules and it has just two dominant alleles (*01:01 and *01:03) distributed with almost identical frequencies (Iwaszko and Bogunia-Kubik 2011). HLA-E maintains a specific and unique role in modulating immune response: it can interact either with the activating CD94/NKG2C or the inhibitory CD94/NKG2A receptor. Structural studies have revealed that both human inhibitory NKG2A and activating NKG2C receptors possess sequences identical in over 75%, however, inhibitory CD94/NKG2A receptor binds to the HLA-E with approximately six-times higher affinity than CD94/NKG2C (Dissociation constant, KDs ranging from 0.7 μM to ∼20 μM for NKG2A and KDs ranging from ∼4 μM to > 0.1 mM for NKG2C) (Kaiser et al. 2005; Valés-Gómez et al. 1999). Variations in the residues forming part of heterodimer interface (165–168 in NKG2C and 167–170 in NKG2A) were found to be responsible for this affinity disparity (Kaiser et al. 2008). Both CD94/NKG2A and CD94/NKG2C bind to the HLA-E α1/α2 domain by recognizing different, but partially overlapping HLA-E epitopes (Wada et al. 2004). A study investigating the effect of HLA-E, expressed by target cells, on a NKG2A^+^ and NKG2C^+^/NKG2A^–^ NK cell subsets, revealed that basal levels of HLA-E activate NKG2A^+^ cells without impact on activation of NKG2C^+^ NK cells (Kaiser et al. 2005; Valés-Gómez et al. 1999). Additionally, the relationship between NKG2C copy number and the NK-cell compartment was not dependent on the HLA-E dimorphism (Muntasell et al. 2013). Though NKG2A and NKG2C receptors are not simultaneously expressed on peripheral CD56^dim^ NK cells, however, almost all decidual CD56^bright^ NK cells and peripheral CD56^bright^ NK cells with active NKG2C simultaneously expressed NKG2A and inhibitory receptor prevails (Kusumi et al. 2006; Sáez-Borderías et al. 2009).

Summarizing the above, HLA-E upholds the balance of NK cell reactivity, following the principle that if both receptors compete in binding to a specific HLA-E molecule, the inhibitory receptor is the preferable one (Lauterbach et al. 2015). The favoured binding to inhibiting CD94/NKG2A is a crucial feature of monitoring the MHC class I expression on normal cells (Joyce and Sun 2011).

**Fig. 2:**
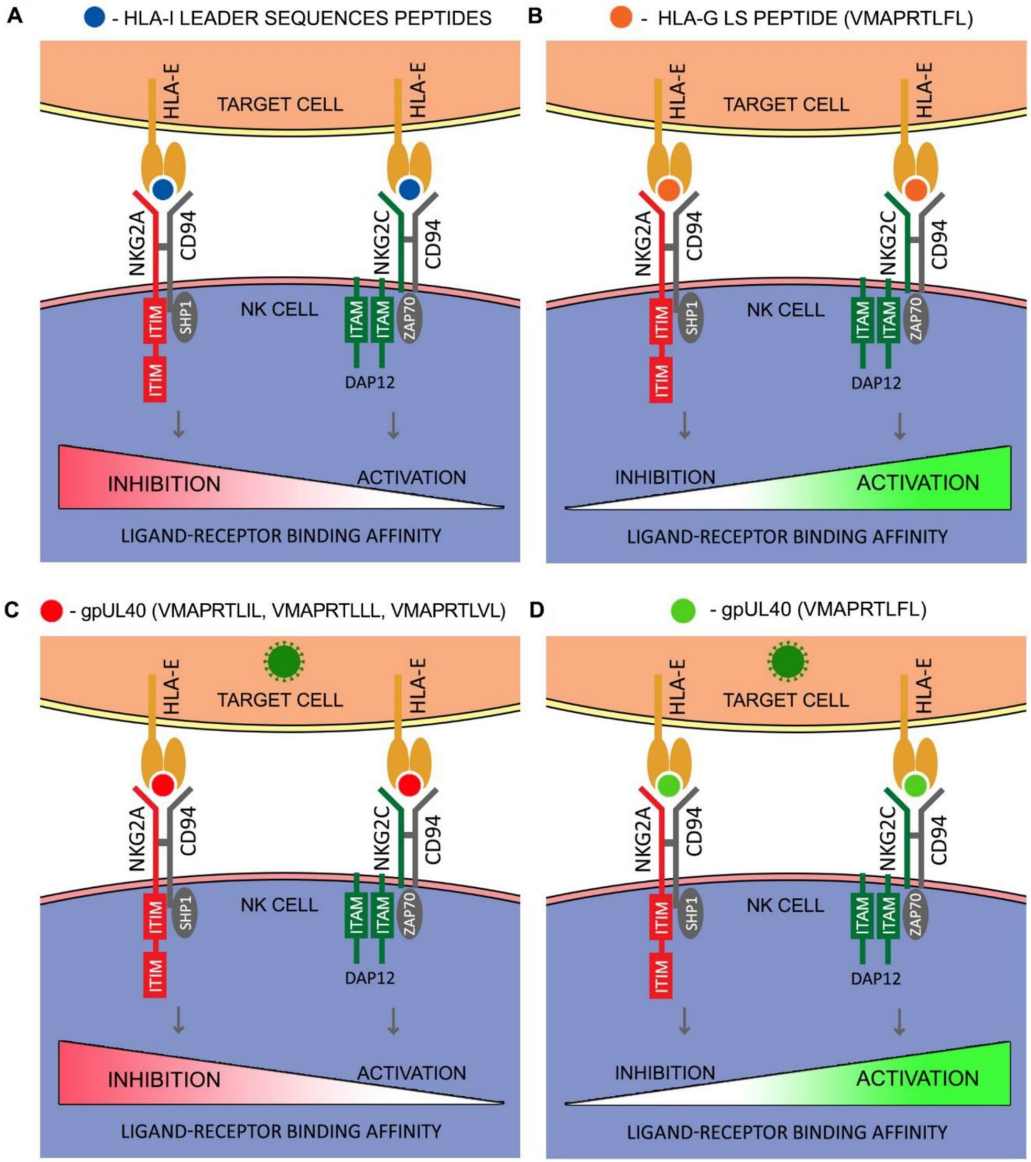
Impact of the peptide presented by HLA-E on balance between NKG2C and NKG2A contradictory signals. Under normal conditions, with basal levels of HLA-E, the NKG2A/CD94 complex is activated by a broad set of peptides derived from the HLA-I leader sequences. This represents a monitoring mechanism for controlling MHC class I expression on normal cells (**A**). When HLA-E is loaded with VMAPRTLFL peptide from the HLA-G leader sequence, the binding affinity of such complex is higher for the activating CD94/NKG2C than for the CD94/NKG2A. Activation signal might cause internalization and degradation of the receptor, preventing excessive NK cell reactivity (**B**). During HCMV infection, HLA-E presents peptides derived from viral gpUL40 which mimic HLA I leader sequences and induce HLA-E expression. Most frequently occurring strains expressed VMARPRTLIL, VMARPRTLLL and VMARPRTLVL with higher affinity for NKG2A, avoiding therefore NK cell attack (**C**). VMAPRTLFL peptide from rare HCMV strain is identical to HLA-G peptide and is sufficient for inducing effector functions in NKG2C^+^ NK cells without co-stimulatory signals (**D**)

Under normal conditions, HLA-E presents conserved peptides derived from HLA-I leader sequences (Fig. 2A). HCMV, like other viruses, evolved a strategy to avoid NK cell killing in HLA I class down-regulated conditions. Virus protein gpUL40 delivers a peptide that mimics leader sequences and binds efficiently to HLA-E molecule inducing its expression (Prod'homme et al. 2012; Tomasec et al. 2000; Ulbrecht et al. 2000). Sequencing of viral UL40 DNA from HCMV isolates provided data about heterogeneity in the repertoire of UL40 peptides (Hammer et al. 2018; Heatley et al. 2013). Mutations in UL40 have an impact on NK cell activation, and remain under host immunological pressure. Most frequently occurring strains expressed VMAPRTLIL, VMAPRTLLL and VMAPRTLVL sequences. The HLA-E/VMAPRTLIL complex is recognized by both NKG2A and NKG2C, however, binding affinity for NKG2C is six-fold lower than for NKG2A (Heatley et al. 2013). In vitro analysis revealed a gradient in activating properties of UL40 peptides on a subset of NKG2^+^C NK cells (Hammer et al. 2018). The most frequently occurred peptides have low affinity for NKG2C, while the rare VMAPRTLFL peptide (identical to HLA-G derived signal peptide) is sufficient for inducing effector functions in NKG2C^+^ NK cells without co-stimulatory signals like interleukin (IL)-12 and IL-18. Viral infections also hamper transporter associated with antigen processing (TAP)-mediated peptide transport. HLA-E can be, therefore, loaded with broad set of unusual peptides with binding motif similar to that of HLA-A*02:01 (Lampen et al. 2013), up to 15 amino acids in length (Hò et al. 2020).

More detailed research have demonstrated further impact of the peptide presented by HLA-E on the balance between NKG2C and NKG2A contradictory signals. The CD94/NKG2A complex has a wide range of interacting peptides resulting in inhibition of NK cells (Lauterbach et al. 2015). In contrast, CD94/NKG2C is regulated by HLA-E loaded with restricted peptide repertoire (Fig. 2B). One of best studied is the nonameric peptide (VMAPRTLFL) derived from the signal sequence of HLA-G. Other peptides that have higher binding affinity for NKG2C than for NKG2A, but to a lesser extent are: HLA-B27 leader peptide (Prašnikar et al. 2021), A80 (VMPPRTLLL), B13 (VTAPRTLLL) (Lauterbach et al. 2015), B7 (VMAPRTVLL), B58 (VTAPRTVLL), Cw3 (VMAPRTLIL), Cw4 (VMEPRTLIL), Cw7 (VMAPRALLL) (Valés-Gómez et al. 1999). However, the in vitro studies showed that HLA-B2705 and HLA-Cw0702 proteins cannot be considered as efficient (Llano et al. 1998; Navarro et al. 1999). UL40 induces surface expression not only of the HLA-E, but also of the gpUL18, a HCMV-encoded HLA-I homologue (Prod'homme et al. 2012). This protein is capable of forming complexes with β_2_-microglobulin (Browne et al. 1990) and endogenous peptides (Fahnestock et al. 1995), including UL40-derived sequences. The surface plasmon resonance-binding studies demonstrated that the gpUL18 weakly binds to the CD94/NKG2C but shows no interaction with CD94/NKG2A receptor in vitro (Kaiser et al. 2008). This finding highlighted a potential role of the CD94/NKG2 receptors in direct responses to the virally encoded proteins, like HCMV gpUL18.

HLA-E presenting HLA-G nonamer binds to the NKG2 receptors with the highest affinity of any others combination tested (Kaiser et al. 2005, 2008; Lauterbach et al. 2015; Llano et al. 1998; Valés-Gómez et al. 1999). The HLA-G-derived HLA-E ligand appears to interact preferentially with the activating CD94/NKG2C receptor (Heatley et al. 2013; Llano et al. 1998). HLA-G is expressed in immune-privileged tissues and in virally (e.g. HCMV) infected cells (Onno et al. 2000; Yan et al. 2009), and, as report by Hò et al. (2020), it is recognized by the NKG2A, as well as ILT2 and KIR2DL4. It plays an important role in pregnancy, transplantations and malignancies (Hò et al. 2020), locally inducing immunological tolerance. The HLA-G level increases when exposed to cytokines (such as interferon (IFN)-γ, IL-10 or transforming growth factor-β), hypoxia or heat stress (Jasinski-Bergner et al. 2022). On the other hand, interaction of HLA-G with NKG2C might cause internalization and degradation of the receptor (Lauterbach et al. 2015). This downregulating mechanism might be another one that prevents excessive NK cell reactivity.

Peptides with activating potential on NK cells should cause a destabilization CD94/NKG2C heterodimer with simultaneous NKG2C and DAP12 communication adjustment (Prašnikar et al. 2021). Molecular dynamics studies revealed that the influential peptide maintains a unique hydrogen bonding network among receptor-ligand “lock and key”-like complex, appropriate for NK cell activation (Prašnikar et al. 2021). Recently, it has been shown that the peptide derived from the non-structural protein 13 of severe acute respiratory syndrome coronavirus 2 (SARS-CoV-2) prevents binding of HLA-E to NKG2A inhibitory receptor, leading target cell to be more susceptible to NK cell attack (Hammer et al. 2022). Therefore, high frequencies of NKG2A^+^ NK cells are found in patients diagnosed with coronavirus disease 2019 (COVID-19), which limits replication of SARS-CoV-2 in lung epithelial cells in vitro. Partially similar mechanism occurs when HLA-E presents peptides derived from hsp60. However, such complex interferes with binding with either NKG2A or NKG2C, making itself unrecognizable to both receptors. This indicates that NKG2 receptors are peptide selective (Michaëlsson et al. 2002).

## NKG2C in Viral Infections

### Human Cytomegalovirus

The NKG2C receptor is well known for its role in many viral infections, especially in HCMV infection. An in vitro study conducted on peripheral blood lymphocytes cocultured with HCMV-infected fibroblasts resulting in expansion of NKG2C^+^ NK cells was the very first evidence of expansion of these cells in response to HCMV infection (Gumá et al. 2006a). This effect was confirmed in many subsequent reports (Gumá et al. 2004; Heatley et al. 2013; Hendricks et al. 2014; López-Botet et al. 2014; Lopez-Vergès et al. 2011; Muntasell et al. 2013), highlighting the critical role of NKG2C in HCMV infection, but from the other side uncovered the impact of HCMV on NK cell subsets composition.

NKG2C^+^C NK cells belong to separated NK cell cluster named adaptive NK cells. Transcriptomic profile of adaptive NK cells derived from bone marrow revealed that not every adaptive NK cell has high NKG2C expression (Yang et al. 2019). When compared to the conventional NK cell subsets, adaptive NK cells displayed a very distinct profile marked by upregulation of *NKG2C, CD3E, PATL2* transcription, downregulation of *CD7, KLRB1* and *FCER1G*, low NKp30, CD161, NKG2A surface expression (Rückert et al. 2022). In HCMV^+^ individuals, adaptive NK cells are characterized by reduction in the expression of FCER1G, ZBTB16, SYK, and EAT-2 compared to other NK subsets (Schlums et al. 2015). Differences in FCER1G, ZBTB16 expression were found between NKG2C^+^ and NKG2C^–^ NK cells subset of HCMV-seropositive patients (Rückert et al. 2022). Epigenetic characteristics of adaptive NK cells depict unique chromatin remodelling within the NKG2 region, which results in *NKG2C* upregulation and *NKG2A* downregulation. Analysis of cis-regulatory elements revealed that adaptive NK cells exhibit increased activity of AP1 motifs, which encode transcription factors involved in defining inflammatory memory in different immune cell types (Larsen et al. 2021).

**Fig. 3:**
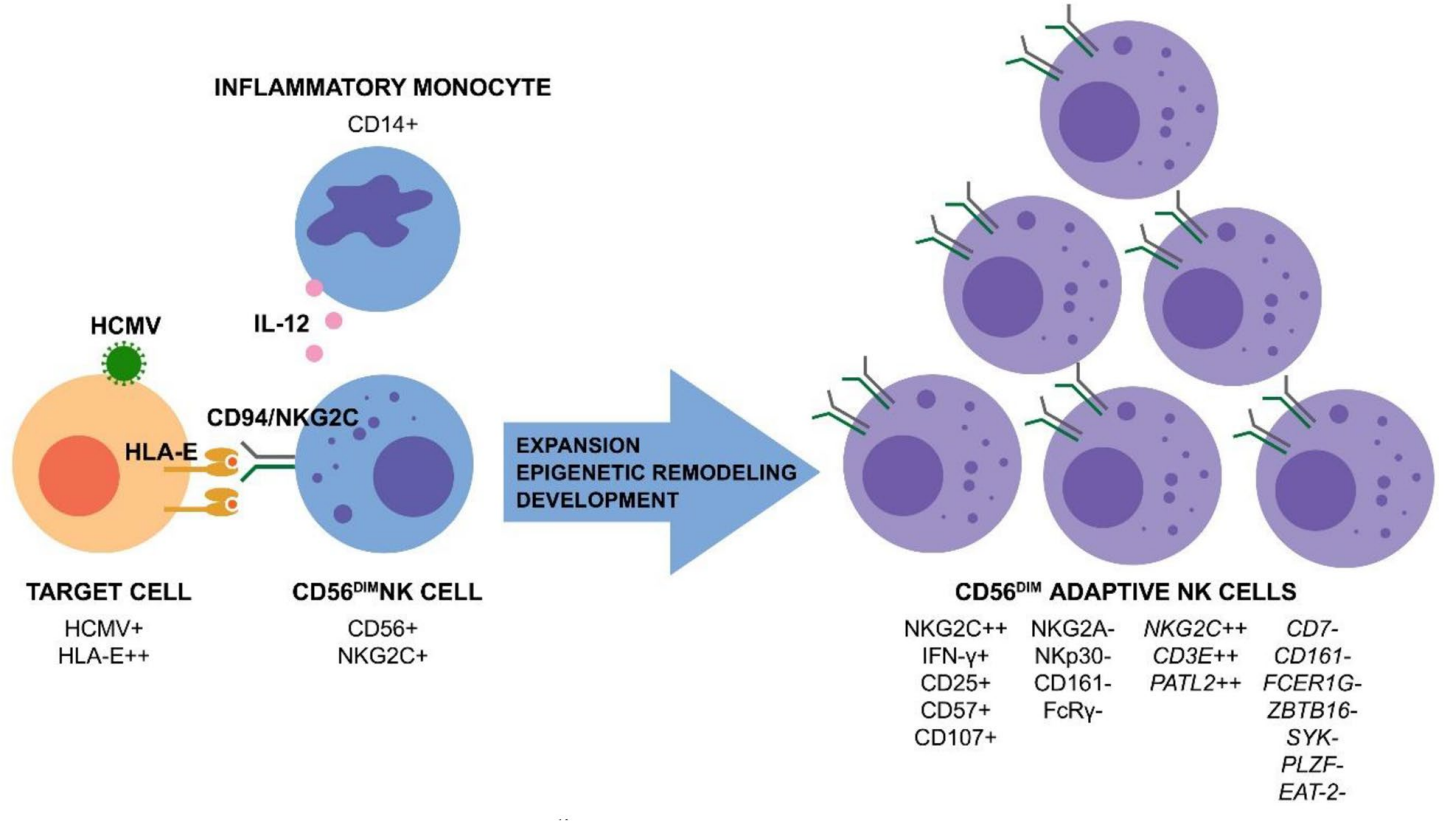
Expansion of adaptive CD56^dim^ NKG2C + NK cells driven by HCMV infection. As a subset of adaptive NK cells, the NKG2C + NK cells display distinct expression profiles when compared to conventional NK clusters

While the expansion of NKG2C^+^ NK cells in the condition of CMV infection seems to be well proven, little is known about the mechanism behind this phenomenon (Fig. 3). There are some examples supporting the idea of NKG2C/CD94 being directly involved in NK cell expansion. In a presence of blocking anti-CD94 monoantibodies, the stimulation of HCMV^+^ donor peripheral blood mononuclear cells (PBMCs) with virus-infected fibroblasts promoted expansion of NKG2C^+^ NK cells and the NKG2C deletion also has influence on this effect (López-Botet et al. 2014). Furthermore, the engagement of proinflammatory IL-12 produced by CD14^+^ monocytes has been regarded as a potential motor agent in NKG2C^+^ NK cells expansion (Rölle et al. 2014). Despite the fact that some clinical observations indirectly suggest that NKG2C^+^ cells contribute to control HCMV replication in vivo, their in vitro response to HCMV-infected cells appears unexpectedly modest and there is no evidence for a triggering role of NKG2C in this system. On the other hand, recent results support that NKG2C^bright^ NK cells are potent effectors of antibody-dependent cellular cytotoxicity (ADCC), and that HCMV-specific antibodies specifically trigger cytotoxicity and cytokine production against infected cells. Remarkably, little information is available regarding the involvement of ADCC in the immune response to HCMV (López-Botet et al. 2014). As they become more mature, the NK cells express more CD57, known as their maturation marker, which is associated with increased cytokine production and ADCC properties (Lopez-Vergès et al. 2010). The CD57^+^NKG2C^+^ NK adaptive cell subset characterises with endurance and resistance to apoptosis. These features correlate with transcriptional changes and epigenetic remodelling, e.g. demethylation of noncoding sequence 1 in the *IFN-γ* gene locus (Tarantino et al. 2021). The presence of HCMV alters the expression of the CD94/NKG2 receptors. It has been proved that HCMV-seropositive adults and children characterise with increased number of NKG2C^+^ cells (Béziat et al. 2013; Heatley et al. 2013; Monsiváis-Urenda et al. 2010). Acute CMV infection promotes high NKG2C^+^ NK cells proliferation, often called NKG2C^(hi)^ NK cells, which eventually leads them to acquire CD57. Such a unique NKG2C^(hi)^ CD57^+^ NK cell subset may be responsible for a specific NK cell memory (Lopez-Vergès et al. 2011). Interestingly, these cells were found to respond specifically to HCMV, but not to Epstein-Barr virus (EBV) infection (Hendricks et al. 2014). NK cells are believed to control the viral infection in the absence of T cells. The NKG2C^+^ lymphocytosis in a patient suffering from acute HCMV infection coincided with a significant reduction of viremia, when the T cells were absent (Kuijpers et al. 2008). The HCMV seropositivity status increases the numbers of both NKG2C^+^ NK and T cells (Muntasell et al. 2013). The NKG2C^+^ NK cells may also regulate the CMV-specific CD8 T cells, which express the HLA-E ligand. In the CMV seropositive individuals, the expansion of CMV-specific CD8 T cells is negatively regulated upon cell activation (Grutza et al. 2020). An in vitro study confirmed that, interestingly, in HCMV-infected monocyte-derived dendritic cells cultures, the endogenous IL-12 secretion affected the NKG2A expression in NKG2C^+^ cells and, therefore, it may be responsible for modulation of the response against HCMV-infected cells. This effect is beneficial for the virus, as the induced expression of CD94/NKG2A strengthens the viral immune evasion mechanisms (Sáez-Borderías et al. 2009). Lack of FcεRγ (FcRγ) expression is another adaptive NK cells marker in response to HCMV. The NKG2C^bright^ and FcRγ^−^ NK cell population was found in HCMV^+^ subjects. Moreover, the loss of FcRγ was accumulated within the NKG2C^bright^ NK cell subset in the *NKG2C wt*/*wt* individuals, and the NKG2C^−^FcRγ^−^ cell population was more frequent in *NKG2C wt/del* and *NKG2C del*/*del* individuals (Muntasell et al. 2016).

Controversially, the recent data suggest that individuals who lack expression of NKG2C show an undisturbed immune response to HCMV infection and the NK cell maturation is not altered by the absence of CD94/NKG2C receptor. This suggests that there are some alternative pathways, which provide similar NK cells activity in case of lacking this receptor (Toson et al. 2022). Besides that, many reports reviewed in the next chapters highlight the key role of the HCMV-induced NKG2C^+^ NK cell expansion observed in other viral infections and inflammatory conditions.

### Hepatitis B and Hepatitis C Virus

The expansion of NKG2C^+^ NK cells in chronic hepatitis patients is associated with an underlying HCMV infection, pointing out the key role of this pathogen in the NK cell activity (Béziat et al. 2012; Malone et al. 2017). Interestingly, there are phenotypic and functional differences in the NK cell repertoire in chronic hepatitis B virus (HBV) infection versus hepatitis C virus (HCV). In case of the HCV infection, the proportion of activated, but more dysfunctional NK cells was higher compared to the HBV-infected individuals. On the other hand, expression of CD94/NKG2C was higher in HBV-infected when compared to HCV-infected and healthy individuals. Additionally, number of circulating NK cells was also lower in HBV and HCV-infected than in control (Oliviero et al. 2009). The NKG2C^+^ NK cell levels can be considered as a prognostic marker of HBV improved treatment responses. The pegylated interferon (PEG-IFN)-α and entecavir are well-known antiviral drugs for HBV treatment. The clinical study conducted on patients suffering from chronic HBV showed that PEG-IFN-α responders have a significantly higher expression of NKG2C^+^ NK cells than non-responders, which suggests the reconditioning and activation of innate immune response during IFN treatment (Yan et al. 2015).

### Human Immunodeficiency Virus

The presence of largely controversial reports obscures understanding the role of CD94/NKG2C receptor in HIV susceptibility, infection and progression. The presence of at least one *NKG2C del*variant was previously indicated as a risk factor of HIV infection (Thomas et al. 2012). The *NKG2C* wt/wt and HLA-E*01:01/*01:01 genotypes were found to be involved in faster and more effective recognition of HIV-infected cells and were found less frequently in long-term non-progressors as well as in HIV-infected patients when compared to controls. Recently published results specify that the NKG2C *del/del* genotype is associated with HIV susceptibility, but only in people living with HIV and not in controls unexposed to HIV or HIV-exposed seronegative subjects (Alsulami et al. 2021; Guzmán-Fulgencio et al. 2013). Unexpectedly, findings from the latest study on HIV-infected Brazilians have questioned any impact of NKG2C genotype on HIV susceptibility (Toson et al. 2022). No association of *NKG2C* deletion with HIV-1 susceptibility or influence on clinical features was observed in the evaluated cohort. The *NKG2C* copy number did not correlate with HIV viral load (Alsulami et al. 2021). Therefore, more genetic studies on larger populations are necessary to better understand this phenomenon.

Unlike other NK cell receptors, the expression of CD94/NKG2C and its ligands is upregulated in HIV patients, thus, the NKG2C may be involved in regulating the infection progress. Many studies indicate that changes in NK receptors repertoire in HIV-1 positive patients are derived from an underlying HCMV coinfection. Advanced stages of HIV-1 disease in patients coinfected with HCMV are characterized by downregulation of CD94/NKG2A and increased expression of CD94/NKG2C (Brunetta et al. 2010; Gumá et al. 2006b; Zhou et al. 2015). This reversed NKG2A/NKG2C ratio is unique for NK cells, but not for CD8^+^ NKG2C^+^ T cells (Zeddou et al. 2007). In a Hispanic case study, high level of memory-like NKG2C^+^ NK cells was associated with the control of HIV-1 viral replication (Climent et al. 2023). However, the high level of NKG2C^+^ NK cells increases the risk of Kaposi sarcoma in patients with HIV-1 infection (Goodier et al. 2007). In summary, the current knowledge of NKG2C^+^ NK cells at mucosal genital/anal sites is still limited, and the NK receptor profile of circulating NK cells may differ from the tissue-resident NK which interact with HIV-infected cells (Alsulami et al. 2021).

### SARS-CoV-2 and COVID-19

There are studies conducted on COVID-19 patients (Jaiswal et al. 2022a; Vietzen et al. 2021a) indicating that *NKG2C* deletion is a risk factor for more severe disease outcome. One of those studies showed that both HLA-E*01:01 and *NKG2C del* variants are more frequently present in hospitalized Austrian COVID-19 patients, and both genotypes are independent risk factors for severe COVID-19 (Vietzen et al. 2021a).

The CD94/NKG2C expression may also be related to the severity of infection. Increased number of circulating NKG2C^+^ NK cells observed in patients suffering from severe COVID-19 was independent of HCMV reactivation and did not correlate with serum levels of anti-CMV IgG. Authors identified upregulated HLA-E in bronchoalveolar lavage (BAL) fluid of COVID-19 patients, and suggested, therefore, a receptor-ligand-driven expansion of adaptive NK cells (Maucourant et al. 2020). Interestingly, even after clearance of SARS-CoV-2, patients with lower recovery of NKG2C^+^ adaptive NK cells were characterized by HCMV reactivation and increased mortality (Jaiswal et al. 2022a). Interesting results were obtained in a study on *Mycobacterium w* (Mw), which is used as an immunomodulator in India. The prophylaxis with Mw used in HCMV-seropositive high-risk cohort resulted in six-fold reduction in incidence of symptomatic COVID-19 over a 6-month period. In a response to Mw, the number of NKG2C^+^ adaptive NK cells increased between 30 and 60 days following treatment. Moreover, the lower baseline of NKG2C^+^ NK cells predisposed to the disease (Jaiswal et al. 2022b). These findings suggest that a higher number of NKG2C^+^ cells is beneficial and provides a protective effect in SARS-CoV-2 infection, whereas a decreased number of these cells, together with the *NKG2C del* variant, weakens it. Interestingly, the NK cell subsets differ in mammalian target of rapamycin activity. Study on rapamycin treatment showed that the percentage of mature NKG2A^−^CD16^+^ CD57^+^ cells significantly increased during treatment and that the level of FcRγ was lower in all NK cell subsets (Shemesh et al. 2022).

### Other Viral Infections

The *NKG2C* deletion did not seem to influence the clinical course of herpetic (herpes simplex type 1 virus) (Moraru et al. 2012), and papillomavirus infection (Vilchez et al. 2013), but predispose to the development of nephropathia epidemica in severe Puumala orthohantavirus cases (Vietzen et al. 2021b). No expansions of circulating NKG2C^+^ NK cells have been observed in studies of patients with recurrent genital herpes simplex virus type 2 infections (Björkström et al. 2011b), likewise in EBV infection in adults (Hendricks et al. 2014). HCMV^+^ EBV^+^ coinfected children had significantly higher proportions of peripheral-blood NKG2C^+^ NK cells than HCMV^+^ EBV^−^ children. These results provide further evidence that the expansion of NKG2C^+^ NK cells is a consequence of CMV-driven immune maturation (Saghafian-Hedengren et al. 2013). NKG2C^+^ NK cell increased expansion was observed in HCMV coinfection with other acute and chronic viral infections: Hantavirus (Björkström et al. 2011a), Chikungunya (Petitdemange et al. 2011), dengue virus-2 (Petitdemange et al. 2016). But on the contrary, results from the latest study on Puumala orthohantavirus indicate that NKG2C^+^ NK cell proliferation and effector functions during virus infections can be also induced independent of prior HCMV infection, for example by the cellular stress response (Vietzen et al. 2021b).

## Pregnancy and Related Complications Affected by NKG2C

Uterine NK cells, accumulated during early pregnancy at the maternal–fetal interface, are engaged in placentation, angiogenesis and regulation of trophoblast invasion (Hanna et al. 2006). It is believed that their dysfunction is a major factor in pathological pregnancies, including pre-eclampsia. The expression of both CD94/NKG2A and CD94/NKG2C receptors is increased on peripheral NK cells from women diagnosed with pre-eclampsia. It might be explained by the scenario in which NK cells try to balance the proper inhibitory/activating receptors’ expression during ongoing systemic inflammation, characterized by high levels of IL-12 and IL-15 (Bachmayer et al. 2009). In accordance, another study revealed expansion of NKG2C^+^ NK cells in pre-eclampsia women. Furthermore, both CD56^–^ and CD56^+^ cell subsets were characterized with increased expression of NKG2A receptor. Women suffering from severe pre-eclampsia had an increased percentage of CD56^bright^ NKG2C^+^ NK cells (Bueno-Sánchez et al. 2013). The role of *NKG2C* deletion in pre-eclampsia susceptibility has been excluded in a Brazilian cohort study (Kaminski et al. 2019).

Although the HCMV causes the majority of intrauterine viral infections, in the first trimester of pregnancy the congenital infection rate is considered to be low. The first trimester is usually the time when the uterus is infiltrated by decidual NK (dNK) cells, which are propitious for placentation. When exposed to HCMV, these dNK cells, normally responsible for chemokines and cytokines production, become cytotoxic for infected autologous decidual fibroblasts. The dNK cells may be involved in controlling the HCMV intrauterine infection and in limiting the viral spreading into fetal tissues. Over 80% of these dNK cells become NKG2C^+^ NK cells (Siewiera et al. 2013). Simultaneously, only a minor decrease of NKG2A^+^ cells was observed. An interesting observation was made in relation to multiple pregnancies. Repeated pregnancies are often related to improved placentation, possibly provided by pregnancy trained decidual NK cells. These cells, involved in vascular remodelling and angiogenesis, are characterized by high expression of NKG2C receptor in HCMV-seropositive women (Gamliel et al. 2018). This observation may lead to question, whether the HCMV serostatus is involved in this NK cells augmentation. Study on endometrium samples from HCMV-seropositive and HCMV-seronegative women showed that endometrial NK cells have an increased pregnancy-induced *LILRB1* expression on NKG2C^+^ cells in HCMV-seropositive women. The HCMV serostatus may be, therefore, involved in the induction of pregnancy-induced memory endometrial NK cells, providing effective placentation (Feyaerts et al. 2019).

The NK cell receptors’ distribution is also altered by the postnatal symptomatic HCMV infection in preterm infants, which is characterized by an early increase of total NK cells, as well as NKG2C^+^ and KIR^+^ NK cell subsets with a simultaneous decrease of the cells expressing CD94/NKG2A receptor (Noyola et al. 2015). Furthermore, in children who had symptomatic congenital HCMV infection, the increased number of NKG2C^+^ NK cells, and lower number of NKG2A^+^ NK cells was found when compared to asymptomatic or non-infected individuals. What is more interesting, this NKG2C^+^ cell increase was associated with *NKG2C wt* genetic variant, and the *wt*/*wt* homozygotes had a higher number of circulating NKG2C^+^ cells than heterozygotes (Noyola et al. 2012).

## Clinical Outcome of Allogeneic Transplantation of Hematopoietic Stem Cells and NKG2C

Hematopoietic stem cell transplantation (HSCT) is a curative treatment for patients whose bone marrow or immune system is damaged or defective. The commonly used clinical practice in allogeneic setting is donor and recipient HLA-typing and determination of the relationship between various alleles and HSCT outcome. Studies show that the relationship between NK cell receptors and their ligands may play a crucial role in the prediction of HSCT outcome (Bogunia-Kubik and Łacina 2021; Isernhagen et al. 2015; Tsamadou et al. 2017, 2019; Yu et al. 2022). The reactivation of HCMV infection remains to be one of the most common post-transplant complications. It is especially threatening to immunodeficient and immunosuppressed patients (López-Botet et al. 2014). NK cells are the first lymphoid subset that can be detected in recipients’ peripheral blood after HSC or cord blood transplantations, however, differences between NK cell development patterns may occur. Patients who experienced the reactivation of HCMV infection after cord blood transplantation characterized with a more rapid NK cell maturation, and expression of NKG2C^+^ NK cells was increased in comparison to patients without reactivation (Della Chiesa et al. 2012).

Interestingly, the early reactivation of HCMV infection is related with lower risk of relapse in acute myeloid leukaemia (AML) patients, unleashing the graft-versus-leukaemia properties. This therapeutic effect was observed similarly in patients diagnosed with chronic myeloid leukaemia, under Dasatinib treatment, where the expansion of NK cells is related to HMCV reactivation (López-Botet et al. 2014). Reduced risk of leukaemia relapse may also be associated with expansion of CD56^dim^CD57^+^NKG2C^+^ NK cells. HSCT recipients treated with reduced-intensity conditioning, who developed HCMV reactivation, were characterized with low relapse rate and better disease-free survival when compared to recipients typed as HCMV-seronegative, suggesting the major role of adaptive NK cell in response to infection (Cichocki et al. 2016; Muñoz-Cobo et al. 2014). Development of HCMV replication after allogeneic HSCT was proven to be a strong and independent predictor of a reduced leukemic relapse risk, and increased long-term survival in early and advanced stages of AML (Elmaagacli et al. 2011).

Besides the altered expression, the CDD94/NKG2C phenotype is also a major factor in the development of post-transplant complications. The *NKG2C del* variant in cord blood transplant recipients increases the risk of HCMV reactivation. In recipients without HCMV reactivation, the wild-type variant was dominant (Mehta et al. 2016). *NKG2C wt/wt* homozygosity was beneficial to the reconstitution and anti-HCMV function of adaptive NKG2C^+^ NK cells. By promoting the quantitative and qualitative reconstitution of adaptive NKG2C^+^ NK cells, donor *NKG2C wt/wt* homozygosity contributes to the clearance of HCMV infection after haploidentical allogeneic HSCT. Thus, considering HCMV infection risk in haploidentical HSCT, *NKG2C* wild-type homozygous donors may be preferable for hematopoietic transplantation (Yu et al. 2022). The donor’s *NKG2C* copy number may be also used as a prediction tool for reactivation of HCMV in double umbilical cord blood transplantation. In recipients receiving two grafts, whose donors carried the deletion variant, risk of reactivation was significantly higher and the reactivation usually occurs within first 3 months after transplantation (Cao et al. 2018).

In HCMV-positive HSCT recipients, the upregulation of NKG2C receptor was observed despite the serostatus of their donors. However, there was a relationship between the HCMV donor status and functionality of NKG2C^+^ NK cells, suggesting that cells transplanted from seropositive donors were more reactive in response to HCMV reactivation, thanks to their previous exposure to HCMV antigens. This indicates the possibility of NKG2C^+^ memory-like NK cells being transplantable (Foley et al. 2012b). In recipients undergoing allogeneic HSCT, reactivation of HCMV infection may induce a graft-versus-leukaemia effect as the donor-derived NK cells eliminate residual leukemic cells. The infection results in increased expression of activating CD8^+^ T cells and NK cells ligands (Foley et al. 2012a). Children undergoing allogeneic HSCT who are diagnosed with acute leukaemia were characterized with reduced risk of relapse and extremely high relapse free survival when both recipient and donor was HCMV-seropositive before transplantation (Behrendt et al. 2009). In contrast to the T cells, NK cells are proven to mediate limited graft-versus-host reactions which gives an opportunity to use a ligand–receptor mismatch between recipients’ residual tumour cells and the NK cell-repleted grafts (Picardi et al. 2015).

A graft-versus-host disease (GvHD) is another common post-transplant complication. In compliance with the natural NK cell maturation, expression of the CD94/NKG2A continuously decreases, whereas expression of CD94/NKG2C increases. A simultaneous increase in NKG2A^+^ and decrease in NKG2C^+^ subsets of CD56^dim^ NK cells was observed in recipients diagnosed with chronic GvHD and in patients with EBV reactivation. A reverse effect occurred in patients without chronic GvHD (Jaiswal et al. 2020; Kordelas et al. 2016). The EBV infection promotes the expression of NKG2A^+^ cells, in contrast to HCMV infection (Hendricks et al. 2014). It has been recently shown that the number of NKG2A^+^ NK^dim^ cells increased with simultaneous decrease of NKG2C^+^ NK^dim^ cells in patients with EBV reactivation and incidence of chronic GvHD after HSCT (Jaiswal et al. 2020). Such alterations in NK cell subsets may increase the risk of chronic GvHD occurrence among HSCT recipients. The activating NKG2C and NKG2D receptors may overcome the impact of highly expressed NKG2A providing a beneficial role in monitoring infections in immunosuppressed patients. Both NKG2 activating receptors were simultaneously upregulated in HSCT recipients 30 and 90 days after transplantation (Picardi et al. 2015). An intriguing case study suggested that the HCMV reactivation may increase the number of NK cells expressing NKG2C receptor. It was observed that in a female recipient of umbilical cord blood transplantation the NKG2C^+^ NK cells constituted one-third of the total lymphocytes for 22 months, after the reactivation of HCMV infection (Muta et al. 2018).

## NKG2C in Organ Transplantation

The HCMV infection and reactivation is a significant clinical problem in lung transplantation, observed in approximately 50% of recipients. Controversially, it was proposed that the increased number of NKG2C expressing NK cells did not provide protection from HCMV reactivation, but increased a risk of viremia. A high proportion of these cells coincided with actively replicating HCMV in lung allograft. Level of the BAL NKG2C^+^ NK cells increased compared to peripheral NKG2C^+^ NK cells, suggests that the CMV infection may drive local changes in the phenotype as well as recruitment of those cells to the transplanted tissue (Harpur et al. 2019). Another study highlights that NKG2C^+^ NK cells prevailed in BAL fluid and were more mature than NKG2C^−^ NK cells. However, in comparison to PBMC NKG2C^+^ NK cells, the BAL NKG2C^+^ NK cells were less mature and proliferative. These findings imply the potential role of the CD94/NKG2C receptor and NKG2C^+^ as biomarkers of HCMV allograft replication and immune activation (Calabrese et al. 2019). In immunosuppressed kidney transplant recipients, highly prone to severe post-transplant complications, the increased level of NKG2C^+^ NK cells before transplantation lowers risk of HCMV reactivation, especially in wild-type homozygous patients (López-Botet et al. 2017). In renal transplantation, recipients with *NKG2C wt*/*wt* genotype were characterized with higher carotid intimal media thickness and lower humoral and T cell responses to HCMV, comparing to *wt*/*del* heterozygotes. This implies that the NKG2C gene deletion, manifested as decreased receptor expression, improved the cardiovascular health of recipients (Waters et al. 2017). Additionally, the pre-transplant NKG2C^+^ NK cells level was associated with post-transplant HCMV viremia. In recipients with high number of NKG2C^+^ NK cells before transplantation, the risk of post-transplant viremia was lower. Thus, it is possible that adaptive NK cells might provide a protection against reactivation of HCMV infection (Redondo-Pachón et al. 2017). From a practical standpoint, monitoring basal and post-transplant levels of adaptive NK cells may provide biomarkers to evaluate the control of HCMV, with viable implications in the clinical management of the viral infection.

## Role of NKG2C in Autoimmune and Other Diseases

Given that CD94/NKG2C is an activating receptor that increase NK cytotoxicity, secretion of proinflammatory cytokines, recruitment of macrophages and other inflammatory cells, it is reasonable to investigate the impact of CD94/NKG2C in autoimmune diseases. The proper control and balance between activating and inhibiting signals seems to be pivotal in autoimmunity development. Experiments conducted on murine models showed, that dysfunction in NK activation (e.g. *NKG2C* deletion) may lead to inability to eliminate autoreactive T cells.

### Rheumatoid Diseases

In rheumatoid arthritis (RA), NK and T cells are responsible for cytotoxicity and cytokine production. NK and T cell activating receptors, including NKG2C, may play an important role in the pathogenesis of this disease. Synovial NK cells characteristic is similar to peripheral blood NK cells, but they express higher number of activation markers. However, considering only the NKG2C expression level, there are no significant differences between synovial and peripheral blood NK cells obtained from patients with chronic joint inflammation, suffered from arthritis of the knee (de Matos et al. 2007). Lower number of NK cells expressing CD94/NKG2C when compared to cells expressing NKG2A was observed in ankylosing spondylitis (Cauli et al. 2018). In autoimmune diseases, the number of specific peripheral lymphocytes, the γδ T cells, is frequently increased (Paul et al. 2015). Detailed expression analysis showed that the NKG2C receptor was more frequently expressed on γδ1 T than on γδ2 T cells in RA patients (Iwaszko et al., submitted). No significant association was observed between homozygous *NKG2C* deletion and RA and systemic lupus erythematosus in Dutch and Japanese patients (Hikami et al. 2003). Conversely, one of the SNPs, the *NKG2C* c.305C > T (Ser102Phe) was shown to be associated with risk of RA in Korean population study (Park et al. 2008). NKG2C c.305*T allele was associated with Behcet’s disease evidencing ocular lesions and Behcet’s disease with arthritis (Seo et al. 2007).

### Psoriasis

To explain the possible association between CD94/NKG2C and HLA-E in psoriasis, two models have been proposed. First, the *NKG2C* deletion together with HLA-E*01:01 genotype is responsible for inhibition of NK cells ability to regulate the autoreactive T cells, which are predisposing to this disease. The *NKG2C del*/*del* genotype was found to be a risk factor in psoriasis susceptibility, which stays in line with the hypothesised model (Zeng et al. 2013). The other explanation considers the impact of HLA-E*01:03 genetic variant. The *01:03 allele alters the presentation of the psoriasis-inducing self-determinant by HLA-C, which provides the protection against psoriasis. This protection can also be provided by the high number of NKG2C^+^ NK cells. Cells expressing this receptor may be able to recognize and then kill autoreactive T cells, however, psoriasis patients characterise with overall decreased number of NKG2C^+^ NK cells, in favour to NKG2A^+^ NK cells. This results in limited regulatory functions of the NK cells which leads to unregulated autoreactivation of T cells (Batista et al. 2013; Patel et al. 2013).

### Other Autoimmune Diseases

The CD94/NKG2C might be considered as a marker for NK reprogramming in autoinflammatory disorders. In celiac disease, intraepithelial cytotoxic T lymphocytes (CTLs) undergo genetic reprogramming that results in acquiring NK cytolytic functions, such as expressing C94/NKG2C receptor. The NKG2C expression by celiac intraepithelial CTLs appeared to be a marker for a general NK reprogramming (Meresse et al. 2006). Patients suffering from multiple sclerosis (MS) display HLA-E^+^ oligodendrocytes and NKG2C^+^CD4^+^ T cells, therefore, targeting the CD94/NKG2C receptor may be considered in future development of MS therapies (Zaguia et al. 2013).

### Other Various Disorders

According to some reports, HCMV-driven NKG2C^+^ NK cell expansion may be a potential predictor for the development of high-risk carotid atherosclerotic plaques (Martínez-Rodríguez et al. 2013). This strands in line with the assumptions that infections may be involved in immunopathology of atherosclerosis. HCMV replicates and remains latent in the endothelial cells, which are constantly exposed to circulating NK cells, triggering NKG2C^+^ NK cell degranulation ex vivo (Djaoud et al. 2016).

Increased proportion of NKG2C^+^ NK cells was also observed in patients suffered from chronic obstructive pulmonary disease (Pascual-Guardia et al. 2020), autism spectrum disorders (Bennabi et al. 2019), schizophrenia and bipolar disorder (Tarantino et al. 2021). The common thread of these findings is lack of association between NKG2C expression level and HCMV status in patients. Despite the limited number of studies, it emphasizes the need to investigate thoroughly the mechanisms of NKG2C^+^ NK cell expansion.

## Current Strategies of NKG2C Clinical Applications in Immunotherapies Against Cancer and Virus-Infected Cells

Increasing activity of the CD94/NKG2C receptor remains to be one of the major directions towards enhancing the expansion of adaptive NK cells. Inhibition of CD94/NKG2A expression is an essential element for the expansion of NKG2C^+^ NK cells with more cytolytic activity (Zuo and Zhao 2021). It was also suggested that inhibition of glycogen synthase kinase-3 promotes the expansion of adaptive NK cells. Their expansion increased the survival in a model of ovarian cancer. Clinical trials conducted on different types of cancers combined with monoclonal antibody therapy are currently in progress (Cichocki et al. 2017).

Activating CD94/NKG2C receptor may be used in NK cell immunotherapy for various tumours. It has been reported by Marin et al. (2003) that haematological malignancies and tumour cell lines characterise with higher expression of HLA-E. Moreover, the latent HCMV infection causes an increase of NKG2C-dependent NK cell cytotoxicity in vitro, which was more effective against target cell lines in HCMV-seropositive donors than in HCMV-seronegative donors (Bigley et al. 2016). Such latent HCMV infection results in accumulation of NKG2C^+^ NK cells, which is a beneficial effect and provides efficient NK cell cytotoxicity against tumour cells. These findings were recently confirmed in vivo in COVID-19 patients (Guo et al. 2022). A NKG2C^+^CD122^low^ cells were identified as memory-like NK cells positively correlated with disease severity and accumulated with age. Their presence depends on patients’ CMV serostatus. This cell subset possesses an expression profile characteristic for adaptive NK cells: upregulation of *NKG2C* and downregulation of *PLZF, FCER1G, EAT-2, SYK*. In vitro IFN-α stimulation in peripheral blood samples led to increased IFN-γ and CD107a levels in elderly patients compared to young individuals.

The HCMV serostatus also has an impact on development of de novo tumours in patients after orthotopic liver transplantation. In HCMV-positive patients the expansion of NKG2C^+^ NK cells is disturbed and the production of intracellular tumour necrosis factor-α is increased. In contrast, immature NK cells with high expression of CD94/NKG2A inhibiting receptor and undisturbed production of IFN-γ were detected in patients diagnosed with genitourinary tumours (Achour et al. 2014). In patients diagnosed with myeloid leukaemia, who have exhausted immunity and naturally lack NKG2C^+^ NK cells, the NKG2C “engager” has the potential to generate a strong antitumor response against acute myeloid leukaemia blasts. Such killer engager provides more efficient NK cell cytotoxicity, however, the frequency of NKG2C^+^ NK cells need to be high to accomplish this effect. Use of the CD94/NKG2C will also result in enhanced NK cell responses in comparison to CD16 (Chiu et al. 2021). The cytotoxic profile of NKG2C^+^ NK cells was upregulated against glioblastoma multiforme cells and altering these cells by the HLA-E*spG feeder cells only enhanced this effect (Murad et al. 2022). It was noted that in glioblastoma cell line modified with HLA-E*spG, the NKG2C^+^ NK cells showed significantly increased cytotoxicity towards them, compared to the parental cells.

A chronic adaptive NK cells stimulation through CD94/NKG2C receptor enhanced proliferation and activation of CD3^–^CD56^dim^CD57^+^NKG2C^+^ NK cells with simultaneously increasing expression of the checkpoint inhibitory receptors namely, lymphocyte activation gene-3 and programmed death receptor 1. These stimulated NK cells were dysfunctional towards tumour cells. This proves that NK cells, similarly to CD8^+^ T cells, show exhaustion when exposed to a chronic activation (Merino et al. 2019).

**Fig. 4:**
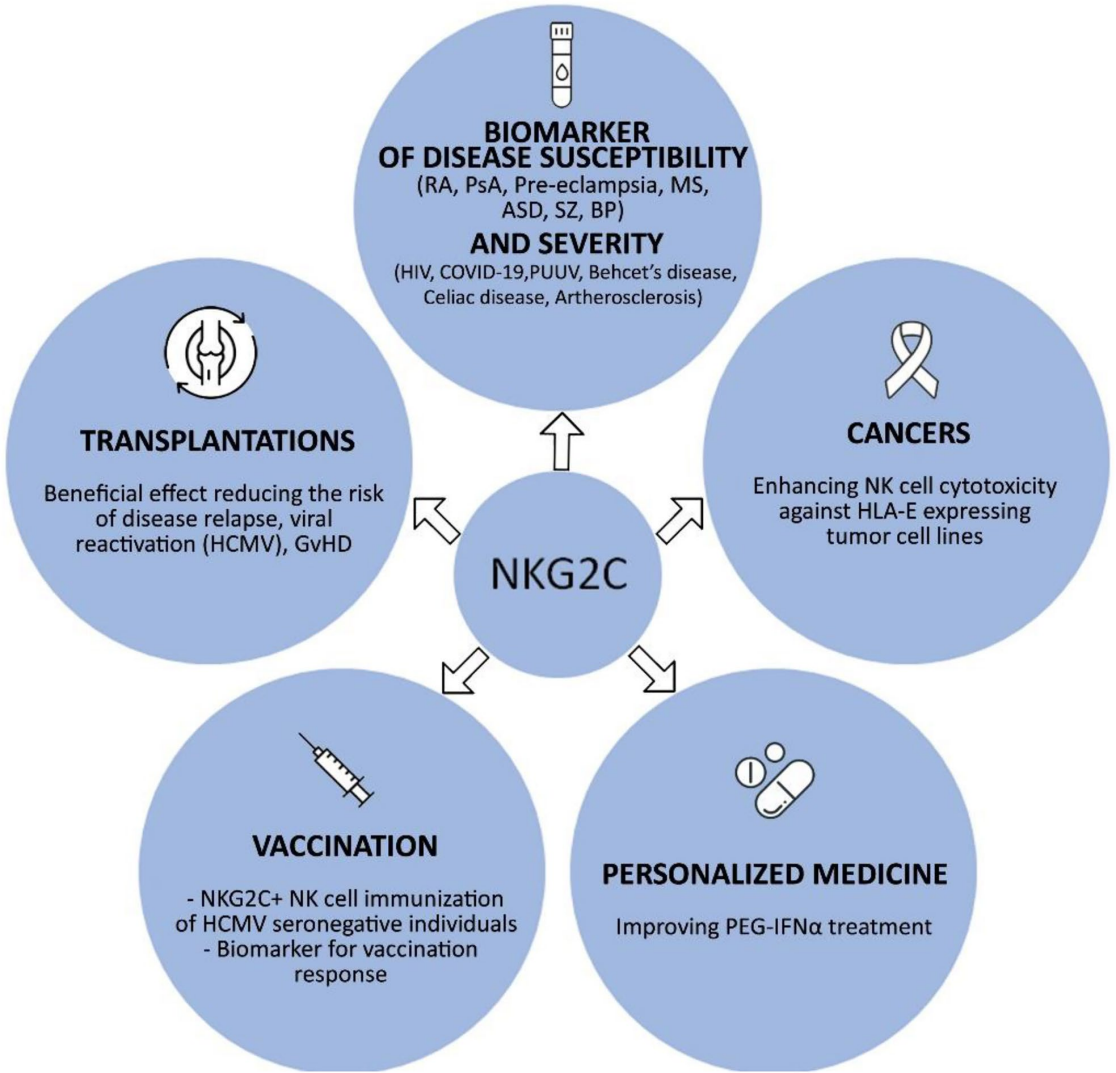
Therapeutic and clinical applications of NKG2C-activating receptor

NK cells are known for interacting with adaptive immune cells directly and indirectly, thus they may be used as indicators of adaptive immunity. The NK cell receptors, including CD94/NKG2C molecule may be crucial in this process. A novel role of the NKG2C as a biomarker in predicting the response to influenza vaccination was suggested in a study conducted on healthy volunteers (Riese et al. 2020). Increased CD56^dim^CD16^+^NKG2C^+^ NK cells frequency was observed in the group of vaccine responders when compared to the group of low responders. Furthermore, the relationship between NKG2C^+^ NK cells and outcome of the vaccination was independent of the patients HCMV serostatus. The CD94/NKG2C receptor acquisition might be used as a marker to distinguish potential low responders from vaccine responders, and promotes efficacious adaptive responses post-vaccination. An interesting approach has been made in studies on HIV vaccine (Gyurova et al. 2020). The non-neutralizing antibodies is a new strategy in vaccine development. The engagement of Fc-binding receptors on NK cells induces release of cytolytic cell compartments and results in death of infected cell. Such cytolytic functions of NK cell together with antibody producing B cells may have a very powerful impact on virus replication. Major areas of the CD94/NKG2C clinical applications are summarised in Fig. 4.

## Conclusions and Further Perspectives

In this review, we investigated biological functions and potential clinical applications of one of the major NK cell receptors, the activating C94/NKG2C molecule. Belonging to the NKG2 receptor family, the CD94/NKG2C remains to be one of the most studied because of its remarkable ability to bind with MHC class I HLA-E molecule, which is a shared ligand for both activating CD94/NKG2C and inhibiting CD94/NKG2A receptors. One of the most common mutations of this receptor results in deletion of its encoding gene, which is not life-threatening but impacting on viral infection, autoimmune diseases, pregnancy, HSCT outcome. In response to exposure to the HCMV viral particles, the expansion of NKG2C^+^ NK cells is increased but detailed mechanism behind this phenomenon remains still not well understood. Thanks to the latest studies, today, we know that these adaptive NK cells undergo phenotypic remodelling (including epigenetic remodelling), acquiring valuable properties for clinical applications. Memory-like NKG2C^+^ NK cells are transplantable, have increased longevity, accumulate with age, possess augmented cytokine release and antibody-dependent cellular cytotoxicity, which all undoubtedly make them inestimable tool for the immunotherapy. The need for further more in-depth research investigating biology of NKG2C^+^ NK cells.is warranted.
